# Deep Sequencing Reveals Direct Targets of Gammaherpesvirus-Induced mRNA Decay and Suggests That Multiple Mechanisms Govern Cellular Transcript Escape

**DOI:** 10.1371/journal.pone.0019655

**Published:** 2011-05-09

**Authors:** Karen Clyde, Britt A. Glaunsinger

**Affiliations:** Department of Plant and Microbial Biology, University of California, Berkeley, California, United States of America; Yale Medical School, United States of America

## Abstract

One characteristic of lytic infection with gammaherpesviruses, including Kaposi's sarcoma-associated herpesvirus (KSHV), Epstein-Barr virus (EBV) and murine herpesvirus 68 (MHV68), is the dramatic suppression of cellular gene expression in a process known as host shutoff. The alkaline exonuclease proteins (KSHV SOX, MHV-68 muSOX and EBV BGLF5) have been shown to induce shutoff by destabilizing cellular mRNAs. Here we extend previous analyses of cellular mRNA abundance during lytic infection to characterize the effects of SOX and muSOX, in the absence of other viral genes, utilizing deep sequencing technology (RNA-seq). Consistent with previous observations during lytic infection, the majority of transcripts are downregulated in cells expressing either SOX or muSOX, with muSOX acting as a more potent shutoff factor than SOX. Moreover, most cellular messages fall into the same expression class in both SOX- and muSOX-expressing cells, indicating that both factors target similar pools of mRNAs. More abundant mRNAs are more efficiently downregulated, suggesting a concentration effect in transcript targeting. However, even among highly expressed genes there are mRNAs that escape host shutoff. Further characterization of select escapees reveals multiple mechanisms by which cellular genes can evade downregulation. While some mRNAs are directly refractory to SOX, the steady state levels of others remain unchanged, presumably as a consequence of downstream effects on mRNA biogenesis. Collectively, these studies lay the framework for dissecting the mechanisms underlying the susceptibility of mRNA to destruction during lytic gammaherpesvirus infection.

## Introduction

Kaposi's sarcoma-associated herpesvirus (KSHV), also known as human herpesvirus 8, is a member of the *Herpesviridae* family of large, enveloped dsDNA viruses. KSHV is associated with a number of malignances, including Kaposi's sarcoma, primary effusion lymphoma (PEL) and multicentric Castleman's disease, primarily in connection with untreated AIDS [1]. KSHV is a member of the *Gammaherpesvirinae* subfamily of herpesviruses, which is comprised of lymphotropic tumor viruses including Epstein-Barr virus (EBV) and a mouse homolog of KSHV, murine herpesvirus 68 (MHV68, also known as murid herpesvirus 4). Like all herpesviruses, gammaherpesviruses undergo both lytic and latent replication cycles. Although latency drives immortalization of infected cells, the lytic cycle is vital for the maintenance of tumors *in vivo*, likely due to the induction of paracrine factors that function in immune evasion, proliferation and angiogenesis [2].

During infection, many viruses dampen cellular gene expression in a process known as host shutoff. Shutoff is believed to serve two main purposes: to block cellular antiviral and stress responses and to reduce competition for limiting gene expression machinery. Viruses achieve shutoff by various mechanisms, including blocking host transcription, mRNA processing, export and translation, and by destabilizing cellular transcripts. During lytic infection, host shutoff by gammaherpesviruses is largely accomplished at the level of RNA stability and is carried out by the alkaline exonuclease (AE) proteins: SOX (ORF37) in KSHV, muSOX (ORF37) in MHV68 and BGLF5 in EBV, likely in conjunction with other cellular factors [3], [4], [5], [6]. All herpesviruses encode an AE, which is critical for viral lytic replication, assisting in the processing of newly replicated viral genomes [7]. However, gammaherpesvirus AEs also demonstrate RNA depletion activity in the cytoplasm [3], [4], [5], [6], a trait not shared by the alphaherpesvirus herpes simplexvirus 1 (HSV-1) AE protein [4], [6]. The active site of the deoxyribonuclease (DNase) activity is also required for ribonuclease (RNase) activity of BGLF5 *in vitro* [8], although the identification of single function mutants that retain DNase activity and lack shutoff and vice versa [3], [9] indicates that additional residues mediate contacts with viral or cellular factors, or the nucleic acids themselves, to specify the substrate.

Previous studies examining the abundance of cellular transcripts during lytic KSHV infection identified a number of mRNAs that appeared to escape host shutoff [10], [11]. However, during infection, additional viral factors regulate transcription, splicing and stability of cellular messages [12], all of which could influence steady state mRNA levels during the lytic cycle. One mRNA in particular, the interleukin-6 (IL-6) transcript, was demonstrated to be refractory to SOX-mediated depletion in the absence of other viral factors [11], indicating that at least some cellular mRNAs are intrinsically resistant to shutoff. In order to assess the impact of SOX on cellular gene expression independent of lytic viral infection, to identify additional potential shutoff escapees, and to characterize for the first time the shutoff induced by MHV68 muSOX, we employed deep sequencing to examine the abundance of cellular transcripts in the presence and absence of SOX or muSOX. Here we show that in agreement with previous studies in cells during lytic KSHV infection [4], [10], [11], there is a dramatic reduction in the levels of most cellular mRNAs in cells expressing SOX and muSOX, with a small percentage of genes induced. Although no obvious commonalities among shutoff escapees were identified, we do detect a strong inverse correlation between transcript abundance and susceptibility to shutoff. Notably, some transcripts that escape SOX are degraded when the cDNA is expressed from a heterologous promoter, whereas other cDNAs are resistant, suggesting that there are multiple mechanisms by which cellular genes can evade host shutoff.

## Results

### SOX and muSOX target most cellular mRNAs in the absence of viral infection

**Figure 1:**
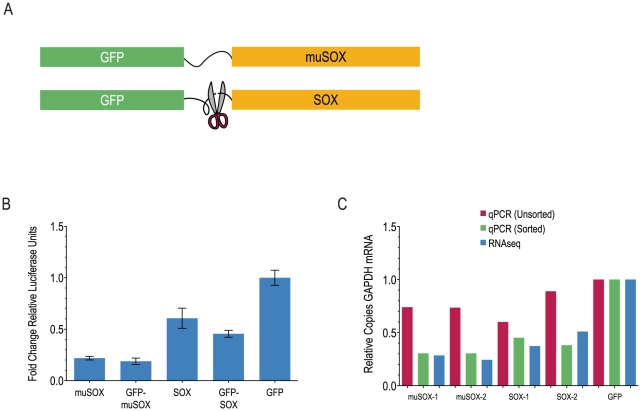
GFP-tagged versions of muSOX and SOX are functional for shutoff. (A) Schematic of shutoff constructs. (B) Co-expression of untagged muSOX, GFP-muSOX, untagged SOX, GFP-SOX and GFP alone with a destabilized luciferase expression construct. Error bars indicate SEM. (C) GFP-normalized levels of endogenous GAPDH mRNA as determined by qPCR on unsorted (red) or sorted (green) cells, or as determined by RNA-seq on sorted cells (blue). Quantitation performed on cells (pre- or post-sorted) used for library construction.

In order to accurately and sensitively quantify the levels of cellular transcripts in the presence of SOX, we first sought to obtain a pure population of SOX-expressing cells. To this end, we generated expression constructs that produce GFP fusion variants of muSOX and SOX. To reduce the chance for interference between the two proteins, a “linker” sequence of 8 glycine residues was inserted between the GFP and muSOX gene coding regions (Fig. 1A). As this strategy did not yield a functional SOX gene (data not shown), an autoproteolytic cleavage site derived from foot-and-mouth-disease virus (FMDV) was inserted between GFP and SOX to yield a GFP with 16 additional amino acids at the C-terminus and SOX with a single added proline at the N-terminus (Fig. 1A). To confirm that host shutoff activity is retained by the GFP fusion proteins, the indicated SOX and muSOX variants were co-expressed with a firefly luciferase reporter construct that contains a destabilizing PEST sequence at its C-terminus (Fluc-PEST), which allows for rapid turnover of fluc protein and thus sensitive quantitation of shutoff. As shown in Figure 1B, the host shutoff activity of GFP-muSOX and GFP-SOX are intact compared to GFP alone, similar to the non-fusion proteins, despite the incomplete cleavage of the GFP-SOX fusion (Fig. S1A). Finally, we confirmed by immunofluorescence microscopy that that GFP with an FMDV-derived C-terminus is functional, and that GFP-expressing cells also express SOX (Fig. S1B).

To generate libraries for deep sequencing analysis, HEK-293T cells were chosen based on their amenability to transfection, the extensive characterization of host shutoff in this cell line, and that 293T support lytic replication of both KSHV and MHV68. Cell monolayers were transfected with the relevant DNA construct and harvested for RNA at 24 h post-transfection. A pure population of GFP-positive cells was isolated by fluorescence-activated cell sorting (488 nm). In order to confirm host shutoff activity in the samples prior to library construction, RNA isolated from unsorted and sorted cells was analyzed for GAPDH mRNA levels by quantitative real-time PCR (qPCR; Fig. 1C). As expected, a greater level of shutoff of the GAPDH transcript is evident in the pure population of GFP+ cells (sorted) compared to unsorted cells. cDNA libraries were prepared from polyA-selected total RNA from GFP-muSOX, GFP-SOX and GFP-transfected cells and subjected to RNA deep sequencing (RNA-seq) analysis on the Illumina platform. Data regarding the depth and quality of the reads are presented in Table 1. Raw reads were aligned to the RefSeq mRNA database using Bowtie with MAC-like rules. Thirty-two nucleotides were discarded from the low quality (3′) end of the read. Counts per gene were normalized to average counts of eight polyadenylated RNA spikes that were introduced into fixed quantities of total RNA prior to polyA selection. Spikes served to correct for variability in RNA and DNA isolation between samples throughout the process of library construction. Correction factors are listed in Table 1. The data indicate a high level of agreement between the abundance of GAPDH in sorted cells as determined by qPCR and as determined by RNA-seq (Fig. 1C).

**Table 1:** Read Data.

	Raw Reads	Filtered Reads (% of Raw)	Unmapped^a^ (% of Filtered)	Spike Correction Factor	Spearman correlation between replicates
**muSOX-1**	18,344,292	13,341,401 (72.7)	2,904,871 (21.8)	1.09	0.9922
**muSOX-2**	18,877,408	13,666,664 (72.4)	3,201,097 (23.4)	1.00	
**SOX-1**	12,401,550	9,912,066 (79.9)	2,533,164 (25.6)	1.77	0.9883
**SOX-2**	14,587,361	11,984,269 (82.2)	1,938,585 (16.2)	2.63	
**GFP**	24,772,163	9,592,286 (38.7)	2,692,482 (28.1)	7.92	

a to RefSeq mRNA database with 32 nt cropped from right end of read.

**Figure 2:**
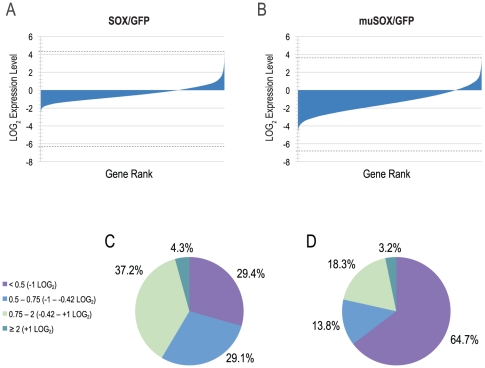
The majority of cellular mRNAs are downregulated in the presence of SOX or muSOX. Expression levels in cells expressing (A) GFP-SOX or (B) GFP-muSOX were normalized to levels in control cells expressing GFP alone, transformed to LOG_2_, then ranked in ascending order and plotted on a linear scale. Dashed lines represent maxima and minima. (C) Pie chart describing the percentages of cellular genes falling into one of the four indicated expression classes in SOX-expressing cells. Purple denotes ≤50% expression (downregulated), blue denotes 50–75% expression (slightly downregulated), light green denotes 75–200% expression (not downregulated or slightly upregulated) and teal denotes ≥200% expression (upregulated), relative to cells expressing GFP. (D) Pie chart as in (C) for muSOX-expressing cells relative to cells expressing GFP.

Reproducibility between replicate samples is high, with a significant correlation between each set (Table 1). To measure the abundance of transcripts in the presence and absence of SOX and muSOX, the average number of spike-normalized reads between duplicate muSOX and SOX samples was compared those in the GFP control sample. Genes were then ranked in ascending order by their expression level relative to GFP control and plotted on a LOG_2_ scale (Fig. 2A and 2B). As with previous observations, MHV68 muSOX was more efficient at inducing host shutoff in 293T cells than its KSHV homolog, SOX [6], [13], with 64.7% of cellular genes downregulated at least 2-fold (−1 LOG_2_) compared to 29.4% with SOX (Fig. 2C and 2D). A higher percentage of genes were moderately decreased (50–75% expression relative to GFP) in SOX-expressing cells than in muSOX-expressing cells (29.1% vs. 13.8%, respectively). Similar to results of previous array studies, which found that approximately 2% of genes were upregulated during lytic KSHV infection [10], [11], 4.3% of genes in SOX-expressing cells and 3.2% in muSOX-expressing cells were induced at least 2-fold. Nearly 42% of genes with SOX and just over one-fifth of genes with muSOX were either not significantly downregulated or were induced (≥75% of GFP; Figs. 2C and 2D).

**Figure 3:**
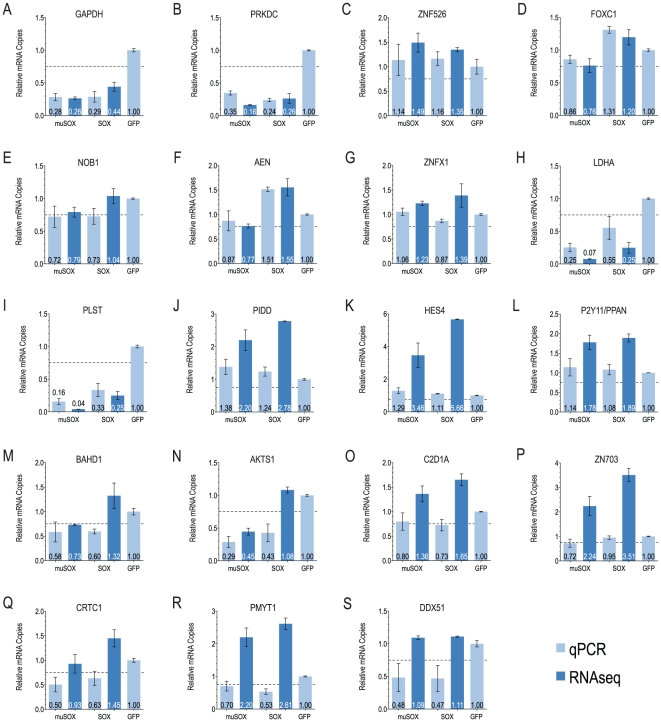
Validation of RNA-seq results by qPCR. Quantitation by qPCR (light blue) or RNA-seq (dark blue) of indicated cellular mRNA levels normalized to levels in cells expressing GFP alone. Error bars for qPCR indicate SEM. Error bars for RNA-seq indicate range. RNA for qPCR and RNA-seq quantitation obtained in separate, independent experiments. Dashed line indicates arbitrary escape cutoff of 75% expression relative to GFP control.

Select cellular mRNAs that were downregulated, upregulated or unaffected by SOX and/or muSOX were validated by qPCR. cDNA was generated from sorted GFP+ cells independently from RNA-seq libraries. Overall, there is good agreement between RNA-seq and qPCR results, though the magnitude of up- or downregulation of mRNAs is in some cases different between techniques. Quantitation of GAPDH by qPCR exhibits close agreement with RNA-seq results (Fig. 3A), as does PRKDC (Fig. 3B), ZNF526 (Fig. 3C), FOXC1 (Fig. 3D), NOB1P (Fig. 3E), AEN (Fig. 3F) and ZNFX1 (Fig. 3G). qPCR of LDHA (Fig. 3H), PLST (Fig. 3I), PIDD (Fig. 3J), HES4 (Fig. 3K) and P2Y11/PPAN (Fig. 3L) validates the overall conclusion of up- or downregulation, with variations in magnitude between RNA-seq and qPCR. qPCR results for BAHD1 (Fig. 3M), AKTS1 (Fig. 3N), CD21A (Fig. 3O) and ZN703 (Fig. 3P) are consistent under some conditions, while CRTC1 (Fig. 3Q), PMYT1 (Fig. 3R) and DDX51 (Fig. 3S) are unreliable for all constructs. Differences in the magnitude and direction of expression change could simply reflect experimental variation or be inherent flaws in either of these techniques for RNA quantitation. GAPDH, which exhibits a high level of correlation between RNA-seq and qPCR quantitation, is one of the most abundant transcripts in cells, whereas other validation targets are far less abundant. It is possible that either or both methods are less reliable for rare transcripts than for abundant ones.

### muSOX and SOX target a common pool of transcripts

**Figure 4:**
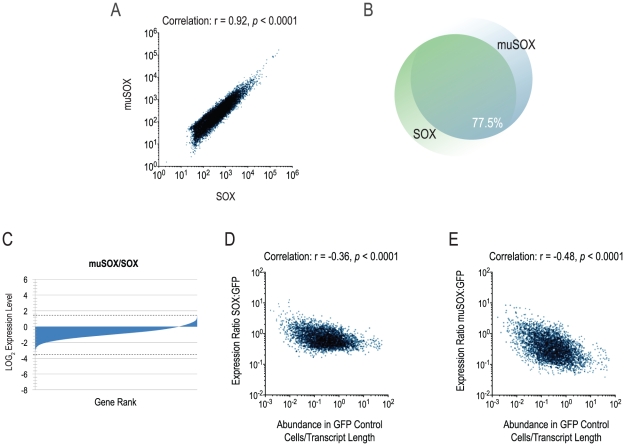
SOX and muSOX target similar pools of host mRNA. (A) Scatter plot of average spike-normalized transcript levels in SOX- vs. muSOX-expressing cells. Correlation calculated by nonparametric Spearman test with indicated r and *p*-values. (B) Venn diagram illustrating overlap in expression levels between SOX- and muSOX-expressing cells. The overlap is calculated from the number of genes that fall into the category of downregulated (<75% expression compared to GFP) or escape (≥75% expression compared to GFP) in both SOX and muSOX-expressing cells. (C) Ranked gene expression in muSOX- vs. SOX-expressing cells. The ratio of expression in muSOX vs. SOX-expressing cells was transformed to LOG_2_ and plotted on a linear scale. Dashed lines indicate maximum and minimum. (D) Inverse correlation between mRNA abundance in control cells and efficiency of shutoff by SOX. X-axis indicates spike-normalized read counts in GFP control cells and y-axis denotes ratio of expression in SOX-expressing vs. GFP-expressing cells. Correlation calculated as in (A). (E) Inverse correlation between mRNA abundance in control cells and efficiency of shutoff by muSOX as in (D).

We have consistently observed that SOX and muSOX function similarly in isolation, mediating mRNA turnover, relocalization of PABPC to the nucleus and hyperadenylation of nuclear mRNA [6], [13]. As this might indicate that SOX and muSOX share a common mechanism of targeting cellular mRNAs, we compared the level of each mRNA in SOX-expressing cells versus muSOX-expressing cells. As illustrated in Figure 4A, there is a strong correlation between the effects of SOX and muSOX on a given mRNA (Spearman r = 0.92, *p*<0.0001). Further indication of the similarity between SOX and muSOX is illustrated by the fact that nearly 78% of mRNAs are similarly regulated by the two shutoff factors (Fig. 4B), in other words, most of the mRNAs that escape shutoff by SOX also escape shutoff by muSOX and likewise for downregulated transcripts. While both factors target similar pools of mRNAs for turnover, muSOX is a more potent shutoff factor in 293T cells, as most cellular mRNAs are less abundant in muSOX- than SOX-expressing cells (Fig. 4C). We next asked whether highly abundant mRNAs, such as those encoding actin and GAPDH, were more susceptible to turnover than rare mRNAs. To this end, we assessed the correlation between mRNA abundance in control cells and their abundance in SOX- or muSOX-expressing cells. As shown in Figures 4D and 4E, messages that are more abundant in control (GFP-expressing) cells tend to be more highly downregulated than rare mRNAs, a characteristic shared by SOX and muSOX. However, the correlation is imperfect, indicating that steady state abundance alone does not dictate a transcript's vulnerability to host shutoff, and suggests that other features of an mRNA play a role. Additionally, any library preparation method is subject to bias, and raw read levels within a sample may not perfectly correlate with abundance.

In order to identify commonalities among genes that are highly downregulated or escape host shutoff, we grouped messages into two categories: shutoff-susceptible and shutoff-resistant. Shutoff-susceptible messages were defined as those present at less that 75% of the level of GFP and escapees as those present at greater than or equal to 75% of the level of GFP. Genes in each class were then assigned major Biological Process gene ontology (GO) terms (Table 2). Although the overall profiles of each expression class (down or escape) were similar, there were several categories that were statistically different between each class. Genes falling into the categories of Metabolic Process, Localization, Establishment of Localization and Cellular Component Biogenesis were overrepresented in the downregulated group, whereas genes of Biological Regulation, Signaling, Multicellular Organismal Process and Developmental Process were enriched among escapees (Table 2). However, attempts to identify more specific ontology terms that were overrepresented among escapees or downregulated transcripts were unsuccessful; in general, most ontology terms and pathways featured a large number of downregulated components and a few escapee or upregulated components with no discernable pattern.

**Table 2:** Distribution of Genes in Major Gene Ontology Terms (SOX/GFP).

Term Name	Term ID	<75% of GFP	≥75% of GFP	*p*-value^a^
Metabolic Process	GO:0008152	2885 (37.1%)	1495 (27.2%)	<0.0001
Establishment of Localization	GO:0051234	1128 (14.5%)	643 (11.7%)	<0.0001
Localization	GO:0051179	1064 (13.7%)	615 (11.2%)	0.0004
Cellular Component Biogenesis	GO:0044085	548 (7.1%)	295 (5.4%)	0.0022
Biological Regulation	GO:0065007	1979 (25.5%)	1584 (28.8%)	0.0006
Signaling	GO:0023052	1169 (15.1%)	952 (17.3%)	0.0133
Multicellular Organismal Process	GO:0032501	781 (10.1%)	728 (13.2%)	<0.0001
Developmental Process	GO:0032502	747 (9.6%)	695 (12.6%)	<0.0001
Cellular Process	GO:0009987	2863 (36.9%)	1892 (34.4%)	ns^b^
Reproduction	GO:0000003	243 (3.1%)	151 (2.7%)	ns
Multi-Organismal Process	GO:0051704	207 (2.7%)	126 (2.3%)	ns
Viral Reproduction	GO:0016032	40 (0.5%)	24 (0.4%)	ns
Pigmentation	GO:0043473	29 (0.4%)	14 (0.3%)	ns
Cell Wall Organization or Biogenesis	GO:0071554	4 (0.1%)	3 (0.1%)	ns
Regulation of Biological Process	GO:0050789	2434 (31.3%)	1827 (33.2%)	ns
Response to Stimulus	GO:0050896	796 (10.2%)	588 (10.7%)	ns
Immune System Process	GO:0002376	286 (3.7%)	243 (4.4%)	ns
Death	GO:0016265	233 (3.0%)	189 (3.4%)	ns
Biological Adhesion	GO:0022610	206 (2.7%)	178 (3.2%)	ns
Growth	GO:0040007	182 (2.3%)	134 (2.4%)	ns
Locomotion	GO:0040011	170 (2.2%)	128 (2.3%)	ns
Rhythmic Process	GO:0048511	34 (0.4%)	35 (0.6%)	ns
Cellular Component Organization	GO:0016043	30 (0.4%)	26 (0.5%)	ns
Cell Killing	GO:0001906	10 (0.1%)	11 (0.2%)	ns
None		2123 (27.3%)	1787 (32.5%)	
Total Genes		7767	5504	

a *p*-value by two-sided Chi-square analysis with Bonferroni correction.

b ns, not significant (*p*>0.05).

### A number of transcripts escape shutoff independent of other viral factors

Previous studies have identified cellular genes that are either targets of or escape host shutoff during viral infection [5], [10], [11]. However, from these studies it was unclear to what extent the downregulation or upregulation was attributable to SOX and what could be a result of infection-specific effects. We examined the genes that were considered escapees in two previous microarray studies and MHC class I genes that were shown to be the targets of EBV BGLF5. With some exceptions, our results are similar, although we were unable to detect the interleukin-6 (IL-6) transcript in our samples. The lack of IL-6 expression in 293T is not surprising as it is induced during KSHV infection by the viral transactivator RTA (ORF50) [14], [15]. Of the HLA class I α-chain paralogs, 22 out of 25 are downregulated, as is the β-2 microglobulin transcript (data not shown). From this we conclude that many of the genes that are downregulated during lytic KSHV infection are likely to be direct targets of SOX.

### ARE-bearing mRNAs are not directly resistant to shutoff

One group of genes that was previously reported to be enriched among escapees during lytic KSHV infection were those whose transcripts bear AU-rich elements (AREs) [10]. AREs are commonly found in highly unstable transcripts of genes that are tightly regulated, such as growth factors and cytokines. AREs are generally destabilizing, but can serve to stabilize messages during a stress response [16]. In SOX-expressing cells, however, we did not find ARE-bearing transcripts to be enriched among escapees, rather, ARE-bearing messages preferentially populated the downregulated group of messages (Table 3). A likely scenario is that many ARE-bearing mRNAs are stabilized independent of SOX during lytic KSHV infection [10], perhaps by the viral protein kaposin B, which has been demonstrated to stabilize transcripts containing AREs by activating the p38/MK2 pathway [17]. Thus, in at least one case, a second viral protein may modulate the shutoff of cellular messages by SOX. Moreover, this illustrates that even highly unstable mRNAs are further destabilized by SOX, and furthermore, that the failure of SOX to deplete select transcripts is not a non-specific effect of enhanced competition for or saturation of the decay machinery [11].

**Table 3:** Abundance of ARE-bearing Messages (SOX/GFP).

Term Name	<75% of GFP	≥75% of GFP	*p*-value^a^: down vs. escape	*p*-value: escapees vs. expected
All AREs	1165 (15.0%)	398 (7.2%)	<0.0001	<0.0001
Class 1 AREs	820 (10.6%)	261 (4.7%)	<0.0001	<0.0001
Class 2 AREs	345 (4.4%)	137 (2.5%)	<0.0001	<0.0001

a *p*-value by two-sided Chi-square analysis.

### Cellular mRNAs utilize multiple mechanisms of escape from shutoff

**Figure 5:**
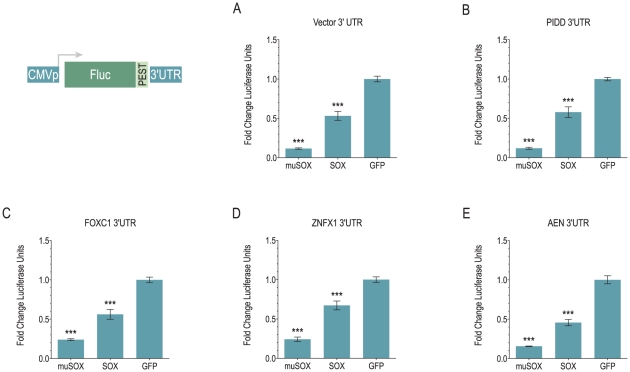
3′UTRs do not mediate escape from shutoff. Translation of destabilized luciferase expression constructs with 3′UTR derived from (A) pcDNA3.1 vector, (B) PIDD, (C) FOXC1, (D) ZNFX1 or (E) AEN in the presence of GFP-muSOX, GFP-SOX or GFP. Raw luciferase values for each construct were normalized to valued obtained in the presence of GFP alone within a given experiment, and error bars indicate SEM. Data are averages of at least 4 independent experiments. *P*-value by two-tailed Mann-Whitney test. ***, *p*<0.0003.

**Figure 6:**
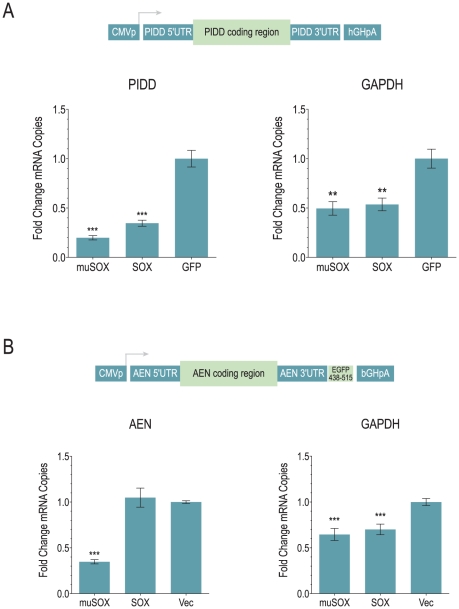
Multiple mechanisms impart protection from host shutoff. (A) Steady-state levels of PIDD transcript expressed from a CMV promoter in the presence of GFP-muSOX, GFP-SOX or GFP. GAPDH levels in the same samples serve as a positive control for host shutoff. (B) Steady-state levels of AEN transcript expressed from a CMV promoter in the presence of untagged muSOX, SOX or empty vector. Data are averages of at least 3 independent experiments. Error bars indicate SEM. *P*-value by two-tailed Mann-Whitney test. **, *p*<0.002; ***, *p*<0.0007.

The lack of discernable patterns among escapees led to us to choose several mRNAs to further dissect, based on their escape from shutoff in both SOX- and muSOX-expressing cells, and their relative abundance in 293T cells to facilitate detection. Since stability and instability elements are most commonly located in the 3′UTR of mRNAs, we cloned the 3′UTRs of 4 genes that fit these requirements, PIDD (LRDD; Fig. 5B), FOXC1 (Fig. 5C), ZNFX1 (KIAA1404; Fig. 5D) and AEN (ISG20L1; Fig. 5E), downstream of a destabilized firefly luciferase gene, and assayed their ability to confer resistance to muSOX and SOX. When co-expressed with muSOX or SOX, 3′UTRs from all four genes were not sufficient to protect the luciferase reporter from shutoff (Fig. 5). Since a few RNA stability regulatory elements have been demonstrated in both the 5′UTR and coding regions of mRNAs [18], [19], we next examined the abundance of the full-length cDNA in cells when expressed from a heterologous promoter. This would negate any direct or indirect effects of host shutoff on transcription and splicing that might account for increased expression during host shutoff. We measured the abundance of transcript from two cDNAs, PIDD and AEN, in cells expressing muSOX or SOX relative to the GFP control. As shown in Figure 6A, the PIDD mRNA is subject to host shutoff when expressed as an intronless transcript from a CMV promoter compared to the endogenous message (Fig. 3J). Similar results were obtained with two other PIDD splicing isoforms (data not shown). GAPDH levels in the same samples were monitored to confirm host shutoff; less robust shutoff of GAPDH here likely reflects the mixed population of transfected and untransfected cells. The levels of ectopic PIDD mRNA in these samples were at least 100-fold higher than endogenous transcript in control cells, indicating that our measurements reflected the CMVp-driven message. In attempting to perform the same experiment with AEN however, we were only able to increase AEN mRNA approximately 2–8-fold over background and thus could not accurately distinguish between effects on endogenous versus CMVp-driven transcripts. To differentiate between the two mRNAs, we expressed the AEN cDNA with a 78-bp sequence from GFP that is recognized by a qPCR assay for GFP (Fig. 6B) [13]. As shown in Figure 6B, unlike the PIDD mRNA, the AEN transcript is resistant to SOX-mediated shutoff, as it is present at similar levels in cells co-expressing SOX as cells transfected with empty vector. In the presence of muSOX, the endogenous AEN mRNA is slightly downregulated (Fig. 3F), though was classified as a potential escapee based on its having 77% expression level in muSOX-expressing cells. The overexpressed AEN is a target of muSOX, perhaps reflecting subtle differences in shutoff activity between the two factors. Similar results were obtained when the AEN transcript (without the GFP amplicon) was expressed from a spliced construct under the EF1a promoter, resulting in more than a 100-fold increase in AEN mRNA compared to the CMVp-driven construct (data not shown), indicating that escape of the AEN mRNA is not simply due to its low abundance in cells.

**Figure 7:**
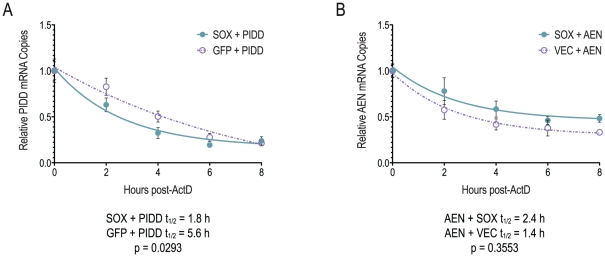
PIDD, but not AEN, is susceptible to degradation by SOX. Half-life of AEN and PIDD mRNAs in the presence and absence of SOX. Cells were transfected with (A) PIDD expression construct and either GFP-SOX or GFP alone or (B) AEN expression construct containing the GFP amplicon and either pCDEF3-SOX or empty vector. At 18 h post-transfection, cells were treated with 2 µg/ml ActD, and total RNA was harvested at the given timepoints. Data are averages of 3 independent experiments. Error bars indicate SEM. *P*-value between rate constants (K) by extra sum-of-squares F test.

As further confirmation that AEN and PIDD mRNAs escape from SOX-mediated shutoff by different mechanisms, we calculated the half-life of each mRNA in the presence of SOX or a control plasmid. 293T cells were transfected with the AEN or PIDD expression plasmid for 18 hours and then treated with ActD to halt nascent transcription. At the indicated timepoints thereafter, remaining AEN or PIDD mRNA levels were determined by qPCR (Fig. 7). As predicted by the steady state levels of exogenously expressed PIDD, its half-life is significantly reduced in SOX-expressing cells compared to GFP control cells (Fig. 7A). In contrast, the AEN mRNA is slightly, though not significantly, more stable in the presence of SOX compared to cells transfected with empty vector (Fig. 7B). Taken together, these data indicate that multiple mechanisms may exist for escape from host shutoff: some mRNAs, including AEN and the previously reported IL-6, are intrinsically resistant to degradation, whereas others may exhibit little change or an increase in steady state levels though the transcript itself is unstable.

## Discussion

Host shutoff is a conserved phenotype of gammaherpesvirus lytic infection [4], [5], [6], [10], [11], [20], [21], [22], and is conferred, in large part, by the alkaline exonuclease homologs of these viruses [4], [5], [6]. Here we have extended previous studies examining the effects of host shutoff during lytic KSHV infection by specifically analyzing the effects of KSHV and MHV68 host shutoff effectors SOX and muSOX, respectively. We demonstrate that while SOX and muSOX have similar effects on cellular mRNAs, muSOX acts as a more potent shutoff factor than SOX at a similar expression level in 293T cells, independent of any regulation by viral genes. Additionally, we detect a strong correlation between basal expression level and degree of shutoff, with more abundant mRNAs being degraded more efficiently. In further characterizing select escapees, we have identified an additional mRNA that is intrinsically resistant to SOX-mediated degradation, AEN. Finally, we have shown that some messages, such as PIDD, are destabilized but exhibit increased steady state levels in SOX- and muSOX-expressing cells, suggesting that there are multiple mechanisms by which cellular mRNAs can evade host shutoff.

The strong inverse correlation between an mRNA's abundance in control cells and the degree to which it is downregulated by SOX or muSOX yields an important insight into the mechanism of host shutoff. This observation suggests that a stochastic means of targeting could be responsible for much of the observed turnover. In support of this model, endogenous PIDD mRNA levels are elevated in SOX or muSOX-expressing cells but the transcript becomes sensitive to shutoff when overexpressed. Alternatively, this could reflect that, as less abundant messages tend to be those that are more tightly regulated, either a dysregulation of accumulation or a regulated stress response to shutoff could be at work. A well known example is the stabilization of ARE-bearing mRNAs by various stimuli [16].

While the correlation between abundance and degradation is strong, it is imperfect, indicating that concentration is not the sole determinant of a transcript's sensitivity and that additional attributes of a given message come into play. Indeed, when AEN (Fig. 6B) or IL-6 [11] transcripts are overexpressed in the presence of SOX, they do not become sensitive to shutoff. At least one gammaherpesvirus shutoff factor has been shown to possess RNase activity *in vitro* [8], although the observed specificity for mRNAs *in vivo* suggests that SOX homologs interface with cellular factors, for example in order to identify the substrate. Whether escapees evade SOX-mediated destruction by virtue of their associated cellular factors or are simply poor substrates for recognition by SOX is a current avenue of investigation in our laboratory.

Another important insight into the mechanisms by which gammaherpesvirus shutoff factors target mRNAs is the discordance between the upregulation of a specific endogenous cellular gene (PIDD) and its abundance when expressed from a heterologous promoter. Perhaps the most plausible explanation is that while the primary function of SOX and muSOX is to induce degradation of mRNAs, dysregulation of transcription and/or processing of cellular messages are secondary effects of host shutoff. For example, endogenous PIDD may escape as a consequence of transcriptional upregulation (perhaps upon depletion of a transcriptional repressor), even though the mRNA may remain susceptible to turnover by SOX, as observed during overexpression. Furthermore, this sort of dysregulation could account for the subset of mRNAs that are more highly expressed in the presence of muSOX relative to SOX, as muSOX may result in even greater damage due its stronger shutoff activity in these cells. If this is the case, it will be interesting to determine whether the virus exploits or compensates for a potentially widespread dysregulation of mRNA biogenesis. Moreover, this may also confound identification of commonalities among “escapees” and could explain our failure to detect shared traits beyond a trend toward degradation of more abundant messages.

We have established that, contrary to lytic KSHV infection, there is no enrichment of ARE-bearing transcripts among those that escape shutoff by SOX alone, suggesting that another viral protein (e.g., Kaposin B) or a cellular response to other aspects of infection (e.g., DNA damage response triggered by genome replication) mediates this effect. Interestingly, both the IL-6 and AEN transcripts, which are not destabilized in the presence of SOX, bear AU-rich elements in their 3′UTRs. Although it is unlikely that these AREs modulate stability during shutoff, there may be other attributes common to the escapee subset of ARE-bearing mRNAs, or highly regulated mRNAs in general, that promote escape from SOX-mediated degradation.

Although SOX and muSOX target similar pools of mRNAs during shutoff, the mechanism of AEN transcript escape appears to be distinct for each factor. The reason for this difference is unclear. It is possible that AEN escapes destabilization in the presence of both factors when it is present at low levels, but at high levels becomes sensitive to the stronger shutoff factor muSOX, due to a slightly different mechanism of targeting. Alternatively, endogenous AEN may escape muSOX by the mechanism we propose for PIDD, in which dysregulation of accumulation of normally highly regulated mRNAs counteracts the loss to shutoff, which would not influence expression from a noncellular promoter (CMVp).

Host shutoff during a lytic KSHV infection in telomerase-immortalized microvascular endothelial (TIME) cells as determined by microarray occurs to a greater extent than in 293T cells expressing SOX alone [10], [11]. There are several possible explanations for this observation. First, it is likely that additional viral factors contribute to shutoff in addition to SOX; indeed, other viral lytic genes such as RTA, ZTA and MTA affect transcription, splicing, export and stability of cellular mRNAs in diverse gammaherpesviruses including KSHV [12]. Additionally, one or more other viral factors may be required for maximum shutoff activity by SOX by modulating its function or specifying its targets. Finally, it is possible that host shutoff by SOX occurs to varying degrees in different cell lineages, perhaps as a result of the availability of host factors that participate in SOX-mediated degradation.

Defects of RNA catabolism are implicated in a number of pathologic states [23], including chronic inflammation [24], [25] and autoimmune [26] and neoplastic diseases [27], [28]. Beyond viral biology and the development of antiviral therapies or vaccines, the study of gammaherpesvirus-induced shutoff has the potential to further elucidate cellular pathways that regulate mRNA accumulation, and how disruption of such pathways may contribute to disease.

## Materials and Methods

### Cloning of GFP Fusion, Luciferase Reporter and Cellular cDNA Constructs

ORF37 genes from Kaposi's Sarcoma-Associated Herpesvirus (KSHV) and Murine Herpesvirus 68 (MHV68), known as SOX and muSOX, respectively, were cloned downstream of green fluorescent protein (GFP) in pcDNA3 (muSOX and GFP) or pcDNA3.1(+) (SOX; Invitrogen) between KpnI and XbaI sites. Primer sequences are listed in Table S1. The GFP protein sequence was separated from that of muSOX by a linker consisting of eight glycine residues. GFP and SOX were cloned flanking an autoproteolytic cleavage site from foot-and-mouth disease virus (FMDV) [29]. Firefly luciferase (Fluc) was derived from pGL3-basic (Promega) and fused to a PEST-coding sequence from pD2-EGFP-N1 (Clontech), and ligated into pcDNA3.1(+) between KpnI and NotI sites. Cellular 3′UTRs were amplified from oligo(dT)-primed cDNA from 293T cells and ligated into the Fluc-PEST base construct between NotI and XbaI sites. In cases where an XbaI site was present in the 3′UTR, a compatible SpeI or NheI restriction site was created at the 3′ end of the UTR fragment, thus destroying the XbaI site upon ligation. The full-length AEN cDNA, corresponding to RefSeq sequence NM_022767.2 was cloned between NheI and BamHI sites in pcDNA3.1(+) or between KpnI and NotI sites in pCDEF3. The amplicon from a previously described qPCR assay for GFP mRNA [13] was generated by annealing and extending BamHI-GFP-fwd and NotI-GFP-rvs primers, and the product was inserted downstream of the AEN 3′UTR in pcDNA3.1 between BamHI and NotI sites. The PIDD (transcript variant 3) expression construct in pCMV6-XL5 was purchased from OriGene (SC126225). Expression constructs containing the coding regions of PIDD isoforms 1 and 2 were a gift of Jürg Tschopp at the University of Lausanne, Switzerland [30], [31]. Fusions and linkers were generated by Splicing by Overlap Extension PCR. GFP fusion proteins were tested by co-transfecting the indicated protein with Fluc-PEST into 293T cells. Luciferase activity was measured at 24 h post-transfection using the Steady-Glo Luciferase Assay substrate (Promega) on a Reporter Microplate Luminometer (Turner Designs).

### Cell Lines and Transfections

HEK-293T cells (ATCC) were maintained in Dulbecco's Modified Eagle's Medium (DMEM; Invitrogen) supplemented with 10% Fetal Bovine Serum (Invitrogen). For cDNA library preparation, 293T cells were transfected with the indicated DNA construct using Effectene (Qiagen) according to the manufacturer's instructions. At 24 h post-transfection, cells were sorted on a Dako-Cytomation MoFlo High Speed Sorter at the Cancer Research Laboratory Flow Cytometry Facility at the University of California, Berkeley. For subsequent experiments, 293T cells were transfected with either Effectene or Lipofectamine 2000 (Invitrogen). For half-life studies, 293T cells were transfected for 18 hours followed by treatment with 2 µg/ml actinomycin D (ActD).

### Immunofluorescence and Immunoblots

HEK-293T cell monolayers were transfected with GFP, SOX or GFP-SOX expression constructs and total protein was isolated at 24 h post-transfection in RIPA buffer (50 mM Tris-HCl pH 7.4, 150 mM NaCl, 2 mM EDTA, 1% (v/v) Nonidet P-40, 0.1% (w/v) SDS) containing Complete Protease Inhibitor Cocktail (Roche) and quantified by Bradford assay (Bio-Rad). Replicate samples were separated by SDS-PAGE, transferred to PVDF membrane and probed with Living Colors mouse anti-A.v. monoclonal antibody (Clontech), affinity-purified rabbit anti-SOX polyclonal antibody [6] and HRP-conjugated goat anti-mouse and goat anti-rabbit secondary antibodies (Southern Biotech). For immunofluorescence, HEK-293T cells were plated onto glass coverslips and transfected as above with the GFP-SOX expression construct. At 24 h post-transfection, cells were fixed with 4% formaldehyde in phosphate-buffered saline (PBS), permeabilized with 1% Triton-X-100, 0.1% sodium citrate in PBS and blocked in 10% normal goat serum in PBS. Slides were incubated with affinity-purified anti-SOX rabbit polyclonal antibody [6] and an Alexa Fluor 546-conjugated goat anti-rabbit secondary antibody (Invitrogen), mounted in VECTASHIELD mounting medium with DAPI (Vector Laboratories) and visualized by fluorescence microscopy.

### Preparation of cDNA Libraries and Sequencing

Libraries were prepared according to the method of Marioni et al. [32] with minor modifications. Briefly, RNA was isolated from approximately 2×10^6^ purity-sorted cells using RNA-Bee (Tel-Test), treated with Turbo DNase (Ambion) and subsequently re-isolated with the RNA Clean-Up kit (Zymo Research). Nine µg of total RNA from each sample was supplemented with 10^8^ strands each of eight polyadenylated spikes (ArrayControl, Ambion) and subjected to two sequential rounds of poly(A) selection on oligo(dT) Dynabeads (Invitrogen). mRNA was partially hydrolyzed in Fragmentation Buffer (Ambion), followed by first strand cDNA synthesis with random hexamers using Superscript II (Invitrogen). Second strand synthesis was conducted with DNA Pol I in the presence of RNase H (Invitrogen). End repair was performed with T4 DNA polymerase, Klenow DNA polymerase and T4 polynucleotide kinase (New England Biolabs). To prepare samples for ligation of adapters, adenosine overhangs were added to library fragments with Klenow (3′to5′exo-) polymerase (New England Biolabs). Paired-end sequencing adapters (Illumina) were ligated with the Quick Ligation kit (New England Biolabs). Cleanup between enzymatic reactions was performed using the QIAquick PCR Purification kit (Qiagen). Gel-purification of an approximately 200 bp fragment of each sample was performed using the QIAquick Gel Extraction kit (Qiagen), and this sample was PCR-amplified for 15 cycles with Phusion polymerase (New England Biolabs) with paired-end primers (Illumina). Samples were again gel-purified and verified on an Agilent 2000 Bioanalyzer DNA 1000 chip (Agilent Technologies) at the Functional Genomics Laboratory at the University of California, Berkeley. DNA concentration was determined by PicoGreen fluorometric assay (Invitrogen). Unpaired sequencing with 76-bp reads was conducted on an Illumina Genome Analyzer IIx at the California Institute for Quantitative Biosciences Vincent J. Coates Genomics Sequencing Laboratory. Raw read data will be available from the National Center for Biotechnology Information Sequence Read Archive under the accession number SRA024508.1.

### Read Alignment

Reads were aligned to the *Homo sapiens* RefSeq database (release 37) with the Bowtie short read aligner [33] using default parameters with the exception that 32 bases were trimmed from the 3′ (low quality) end of each read. Genes without at least 50 normalized reads in one or more samples were discarded, as their reproducibility was low. Since Bowtie allocates isoform multireads to transcripts stochastically, we evaluated expression at the gene level rather than at the transcript level. To control for the efficiency of RNA isolation and adapter-tagged cDNA synthesis, raw expression values were normalized to the average abundance of each of the eight spike controls. Spike-normalized expression values were then converted to expression ratios relative to a given gene's expression in cells transfected with GFP alone. To assess the purity of the RNA-seq libraries, the unmapped reads in one library (muSOX-1) were analyzed: most unmapped reads aligned to the human genome under less stringent parameters in Bowtie or BLAST [34]. 68.5% of non-human reads aligned to nothing, possibly representing random events occurring during PCR or sequencing. Most of the remaining reads aligned to orthologs of other mammals, mainly primates, probably due to errors incorporated during library preparation and sequencing. Other reads mapped to adenovirus and SV40 virus genomic sequences, both of which were used in the preparation of the 293T cell line, and to the pcDNA3 plasmid and the genomic DNA of *Escherichia coli*, in which the plasmid was propagated.

### Data Processing and Statistical Analysis

Calculations of RNA-seq and qPCR data were conducted in Excel 2008 for Macintosh (Microsoft). Graphing and statistical analyses were performed in Excel and in Prism 5 (GraphPad Software). Half-life was calculated by one phase decay nonlinear regression in Prism. Genes assigned to various ontology terms were derived from AmiGO Version 1.7 on the Gene Ontology website [35]. The presence, type and number of AU-rich elements for each cellular gene was compiled from the ARED 3.0 database [36]. Mapping of gene identifiers to different annotation systems was performed by ID Converter version 2.0 [37]. UTR and mRNA sequences for purposes of read distribution and comparisons of length and expression level were obtained from Ensembl using BioMart [38].

### Quantitative Real-Time PCR

Total RNA was isolated from cells using the Zymo Mini RNA Isolation II system (Zymo Research), treated with Turbo DNase (Ambion) and subjected to reverse transcription with AMV RT (Promega) in the presence of oligo dT and 18S reverse primers. cDNA levels were quantitated with gene-specific primer/probe sets (Applied Biosystems) specific for human G3P (GAPDH; 402869), LDHA (Hs00855332_g1), PLST (Hs00192406_m1), PRKDC (Hs00179161_m1), PMYT1 (Hs00177774_m1), PIDD (Hs00388035_m1), ZN703 (Hs00228155_m1), HES4 (Hs00368353_g1), P2Y11 (Hs00220301_m1), CRTC1 (Hs00257715_m1), AKTS1 (Hs00260717_m1), FOXC1 (Hs00559473_s1), NOB1 (Hs00758979_sH), BAHD1 (Hs01050936_g1), C2D1A (Hs00214594_m1), ZNFX1 (Hs00397459_m1), AEN (Hs00224322_m1), ZNF526 (Hs00384824_g1) and DDX51 (Hs00403498_m1). The qPCR assay for GFP was conducted as previously described without MgCl_2_ supplementation [13]. qPCR was carried out using the TaqMan Universal PCR Master Mix on a 7300 Real-Time PCR System (Applied Biosystems). To control for efficiency of RNA isolation, reverse transcription and PCR amplification, values were normalized to 18S levels determined in a parallel reaction using TaqMan Ribosomal RNA Control Reagents (Applied Biosystems).

## Supporting Information

Figure S1 **GFP-SOX expression in 293T cells.** (A) Anti-SOX and anti-GFP antibodies were combined to reveal the uncleaved GFP-SOX fusion and processed GFP and SOX. Equal quantities of protein from replicate samples were loaded in each well. (B) Expression of GFP-SOX in 293T cells. Blue, DAPI staining of nuclei; green, GFP; orange, SOX pAb.
(EPS)

Table S1 Primer sequences used for plasmid construction.
(DOCX)
