# Supplementary material for: Deep Sequencing Reveals Direct Targets of Gammaherpesvirus-Induced mRNA Decay and Suggests That Multiple Mechanisms Govern Cellular Transcript Escape
Source: PLoS One. 2011 May 9;6(5):e19655. doi: 10.1371/journal.pone.0019655 (PMC3090416; doi:10.1371/journal.pone.0019655)
Supplement: Table S1 — Primer sequences used for plasmid construction. (DOCX) [file pone.0019655.s002.docx]

**Table S1. Primer Sequences**

| **Construct** | **Primer Name** | **Sequence (5' 🡪 3')** |
| --- | --- | --- |
| GFP-SOX,  GFP-muSOX | KpnI-GFP-fwd | ATTTGGTACCATGGTGAGCAAGGGCGAG |
| GFP-muSOX | GFP-G8-rvs | CCCACCTCCGCCCCCGCCTCCACCAAGCTTCTTGTACAGCTCGTCC |
| GFP-muSOX | G8-muSOX-fwd | GGTGGAGGCGGGGGCGGAGGTGGGGAAGGGTCGATTATTCTGGATTTTTTTG |
| GFP-muSOX | muSOX-XbaI-rvs | ATAGTCTAGAGGCCGCTTAGGGGGTTATG |
| GFP-SOX | GFP-FMDV-rvs | CTCGACGTCTCCGGCAAGCTTAAGAAGGTCGAAGTTAAGCTTCTTGTACAGCTCGTCC |
| GFP-SOX | FMDV-SOX-fwd | AAGCTTGCCGGAGACGTCGAGTCCAACCCTGGGCCCGAGGCCACCCCCACACCC |
| Fluc-PEST | KpnI-Fluc-fwd | TTGGTACCATGGAAGACGCCAAAAACATAAAG |
| Fluc-PEST | Fluc-PEST-rvs | GAAGCCATGGCTCACGGCGATCTTTCCGCC |
| Fluc-PEST | Fluc-PEST-fwd | GATCGCCGTGAGCCATGGCTTCCCGCCG |
| Fluc-PEST | PEST-NotI-rvs | TCGAGCGGCCGCCTACACATTGATCCTAGCAGAAGCAC |
| Fluc-PEST-AEN 3'UTR | NotI-AEN-fwd | AATTGCGGCCGCGAAGGGGGCGGGGCTCCC |
| Fluc-PEST-AEN 3'UTR | NheI-AEN-rvs | GCCGGCTAGCTCACGTTTCCAATTGAGTTTTATTCTAGAAC |
| Fluc-PEST-FOXC1 3'UTR | NotI-FOXC1-fwd | AATTGCGGCCGCCACACCCTCAAAGCCGAACTAAATC |
| Fluc-PEST-FOXC1 3'UTR | NheI-FOXC1-rvs | CGCGGCTAGCTGACTGAAATTAAAGTGTATTTATTTGCAGCAATC |
| Fluc-PEST-PIDD 3'UTR | NotI-PIDD-fwd | ATATGCGGCCGCGCCCCACAGACTTTTAGGCTG |
| Fluc-PEST-PIDD 3'UTR | XbaI-PIDD-rvs | GCGCTCTAGATGAGGTGAAAGAAACAGTGCAGTTTTG |
| Fluc-PEST-ZNFX1 3'UTR | NotI-ZNFX1-fwd | AATTGCGGCCGCGAAGATGGTACACCACTGCCTTTTG |
| Fluc-PEST-ZNFX1 3'UTR | SpeI-ZNFX1-rvs | CCCCACTAGTTCTCAATCATTGTTTTTAATTGGCTTTATAAGC |
| AEN-FL | NheI-AEN 5'-fwd | ATAAGCTAGCCCGGAAGAGACACGCGG |
| AEN-FL | BamHI-AEN-rvs | CCCCGGATCCTCACGTTTCCAATTGAGTTTTATTCTAGAAC |
| AEN-FL | BamHI-GFP-fwd | AATAGGATCCCAACAGCCACAACGTCTATATCATGGCCGACAAGCAGAAGAACGGCATCAAG |
| AEN-FL | NotI-GFP-rvs | TATTGCGGCCGCATGTTGTGGCGGATCTTGAAGTTCACCTTGATGCCGTTCTTCTGCTTGTC |
